# Learning Brain Connectivity Sub-networks by Group- Constrained Sparse Inverse Covariance Estimation for Alzheimer's Disease Classification

**DOI:** 10.3389/fninf.2018.00058

**Published:** 2018-09-07

**Authors:** Yang Li, Jingyu Liu, Jie Huang, Zuoyong Li, Peipeng Liang

**Affiliations:** ^1^School of Automation Sciences and Electrical Engineering, Beihang University, Beijing, China; ^2^Beijing Advanced Innovation Center for Big Data and Brain Computing, Beihang University, Beijing, China; ^3^Beijing Advanced Innovation Center for Big Date-Based Precision Medicine, Beihang University, Beijing, China; ^4^Fujian Provincial Key Laboratory of Information Processing and Intelligent Control, Minjiang University, Fujian, China; ^5^Department of Radiology, Xuanwu Hospital, Capital Medical University, Beijing, China; ^6^School of Psychology, Capital Normal University, Beijing, China

**Keywords:** Alzheimer's disease (AD), resting-state fMRI, Group-constrained topology structure detection, sparse inverse covariance estimation (SICE), functional connectivity network, classification

## Abstract

**Background/Aims:** Brain functional connectivity networks constructed from resting-state functional magnetic resonance imaging (rs-fMRI) have been widely used for classifying Alzheimer's disease (AD) from normal controls (NC). However, conventional correlation analysis methods only capture the pairwise information, which may not be capable of revealing an adequate and accurate functional connectivity relationship among brain regions in the whole brain. Additionally, the non-sparse connectivity networks commonly contain a large number of spurious or insignificant connections, which are inconsistent with the sparse connectivity of actual brain networks in nature and may deteriorate the classification performance of Alzheimer's disease.

**Methods:** To address these problems, in this paper, a new classification framework is proposed by combining the Group-constrained topology structure detection with sparse inverse covariance estimation (SICE) method to build the functional brain sub-network for each brain region. Particularly, to tune the sensitive analysis of the regularized parameters in the SICE method, a nested leave-one-out cross-validation (LOOCV) method is adopted. Sparse functional connectivity networks are thus effectively constructed by using the optimal regularized parameters. Finally, a decision classification tree (DCT) classifier is trained for classifying AD from NC based on these optimal functional brain sub-networks. The convergence performance of our proposed method is furthermore evaluated by the trend of coefficient variation.

**Results:** Experiment results indicate that a LOOCV classification accuracy of 81.82% with a sensitivity of 80.00%, and a specificity of 83.33% can be obtained by using the proposed method for the classification AD from NC, and outperforms the most state-of-the-art methods in terms of the classification accuracy. Additionally, the experiment results of the convergence performance further suggest that our proposed scheme has a high rate of convergence. Particularly, the abnormal brain regions and functional connections identified by our proposed framework are highly associated with the underpinning pathological mechanism of the AD, which are consistent with previous studies.

**Conclusion:** These results have demonstrated the effectiveness of the proposed Group- constrained SICE method, and are capable of clinical value to the diagnosis of Alzheimer's disease.

## Introduction

Alzheimer's disease (AD) is one of the most common neurodegenerative diseases and generally causes impairments in multiple cognitive functions such as memory, attention, verbal, and visuospatial abilities (Alzheimer's Association, 2015). This disease adversely affects the daily life of AD patients, and eventually results in death. Currently, there is no clinical cure method for the AD so far (Alzheimer's Association, 2015). Considering the high incidence of the AD, it is highly required for the precise diagnosis of the AD individually so that some effective behavioral or pharmacological treatment can be adopted to alleviate the symptoms and delay the progression of the AD (Brookmeyer et al., 2007).

Recently, functional connectivity networks constructed from the functional magnetic resonance image (fMRI) hold great promise for distinguishing AD patients from NC (Rosa et al., 2015). Numerous functional connectivity modeling methods have been proposed to investigate brain functional connectivity activities, including correlation-based and sparse representation-based methods (Wee et al., 2014; Meszlényi et al., 2017). For example, Khazaee et al. (2016) constructed the functional connectivity networks for AD classification by using a Pearson correlation-based method and the weak connections were removed by maximizing the global cost efficiency (GCE) in the networks. Their findings demonstrated that the region of interests (ROI) of the Thalamus, Paracentral_Lobule, and Temporal_Pole_Mid are significantly different between the AD and NC. Furthermore, a high-order functional connectivity network was discussed based on the sliding window, the Pearson correlation coefficient and minimum spanning tree method for AD classification Guo et al. (2017). Their experiment results indicated that the functional connectivity between the Precentral and Supp_Motor_Area is the discriminative feature for identifying individuals with the AD from NC subjects. Among these methods, the correlation-based methods generally obtain relatively high sensitivity in detecting network connections due to the negative connections in the connectivity networks. However, correlation-based network analysis can only capture the pairwise information and does not effectively characterize the functional interactions among many brain regions working together (Huang et al., 2010; Li et al., 2014). In addition, correlation-based fully connected functional networks may contain a large number of spurious or insignificant connections, and may deteriorate the classification accuracy and generalization performance of the classifiers. Recent work has shown that robust connections can be elucidated from a set of noisy connections when certain sparsity constraints are imposed on the connectivity networks (Supekar et al., 2008; Zanin et al., 2012). The sparsity constraint is validly correlated with the small-world property, where a single brain region usually only interacts with a small number of other brain regions (Stam et al., 2007; Supekar et al., 2008; Li et al., 2014).

Recently, the sparse inverse covariance estimation (SICE) method has been widely applied to constructing brain functional connectivity and detecting the connectivity difference between patients with neurological diseases and NC (Huang et al., 2010; Rosa et al., 2015; Zhang et al., 2015; Qureshi et al., 2017). For example, Huang et al. (2010) employed the SICE scheme in the positron emission tomography (PET) data to observe brain connectivity difference between AD and NC. Rosa et al. (2015) further combined the SICE with a sparse discriminative classifier (linear *l*_1_-norm support vector machine (SVM)) to discriminate major depressive disorder (MDD) patients from NC. However, the SICE is a sparse constraint method applied at the individual level, inevitably generating different network topologies for different subjects and causing the inter-subject variability (Wee et al., 2014). This deficient may degrade the generalization performance of the classifiers due to the difference of the different functional connectivity among subjects (Wee et al., 2014). Additionally, the SICE method commonly may encounter the overfitting problem due to the limitation of the small sample size, which is a main challenge in computer-aided diagnosis of the AD (Zhang et al., 2018).

To overcome these deficiencies, a new classification framework based the functional brain sub-network is proposed. The key of the proposed framework is that a Group-constrained topology structure detection algorithm is first used to select the most discriminative brain regions, indicating that the connection topology is identical among subjects. Then the SICE algorithm is further employed to preserve the individual information via different connectivity values. Specifically, the Group-constrained topology structure detection algorithm utilizes a *l*_2, 1_-norm penalization term to encourage an identical connection topology among subjects and minimize the inter-subject variability, and thus a better classification performance was achieved (Wee et al., 2014). Furthermore, the sample size requirement of the Group-constrained topology structure detection is much weaker than that of traditional *l*_1_-norm sparse methods (Mitra and Zhang, 2016). Therefore, the proposed Group-constrained connectivity structure detection scheme can alleviate the small sample size problem. Finally, a nested LOOCV scheme is implemented to tune the regularization parameter in the functional brain sub-network construction process and further to evaluate the effectiveness of the proposed framework. Experimental results demonstrate that our classification framework achieved a high cross-validation accuracy of 81.82%, outperforming the competing methods with a relatively large margin. Furthermore, the area under ROC curve (AUC), regarded as a metric of diagnostic power, of 0.9667 demonstrates the efficacy of our proposed framework in extracting discriminative information for diagnosing the AD. Particularly, the abnormal functional connections identified by our proposed framework are highly associated with the underpinning pathological mechanism of the AD, which are in line with previous studies.

The remaining sections are organized as follows. An explicit description is provided for fMRI dataset acquisition and the process of constructing functional brain sub-networks in Materials and Method sections. Then, the classification performances of different brain network construction methods as well as the most discriminative connections are presented in Results and Discussion sections. Finally, we conclude this study in Conclusion section.

## Materials

### Subjects

Sixty-two right-handed subjects (30 AD and 32 healthy controls) participated in this study after giving written informed consent. AD subjects were recruited from patients who had consulted a memory clinic for memory complaints at Xuanwu Hospital, Capital Medical University, Beijing, China. The healthy elderly controls were recruited from the local community through advertisements. This study was approved by the Medical Research Ethics Committee of Xuanwu Hospital, Capital Medical University.

All AD patients underwent a complete physical and neurological examination, standard laboratory tests, and an extensive battery of neuropsychological assessments. The diagnosis of AD fulfilled the Diagnostic and Statistical Manual of Mental Disorders 4th Edition criteria for dementia (Frances et al., 1994), and the National Institute of Neurological and Communicative Disorders and Stroke/Alzheimer Disease and Related Disorders Association (NINCDS-ADRDA) criteria for possible or probable AD (McKhann et al., 1984). The subjects were assessed with the Clinical Dementia Rating (CDR) score (Morris, 1993) as (mainly) being in the early-stages of the AD (2 patients with CDR = 2, 14 patients with CDR = 1 and 14 patients with CDR = 0.5).

The criteria for healthy controls were as follows: (1) no neurological or psychiatric disorders such as stroke, depression, epilepsy; (2) no neurological deficiencies such as visual or hearing loss; (3) no abnormal findings such as infarction or focal lesion in conventional brain MR imaging; (4) no cognitive complaints; (5) MMSE score of 28 or higher; (6) CDR score of 0.

Data from seven subjects (five AD patients and two healthy controls) were excluded due to excessive motion. Thus, the remaining 55 participants were included in the following data analysis. There were no significant differences between the two groups in gender, age, and years of education, but the MMSE scores were significantly different (*p* < 0.001) between two groups.

### Data acquisition and pre-processing

MRI data acquisition was performed on a SIEMENS Trio 3T scanner (Siemens, Erlangen, Germany). Foam padding and headphones were used to limit head motion and reduce scanner noise. The subjects were instructed to hold still, keep their eyes closed and think nothing in particular. Functional images were collected axially by using an echo-planar imaging (EPI) sequence [repetition time (TR)/echo time (TE)/flip angle (FA)/field of view (FOV) = 2,000 ms/40 ms/90°/24 cm, resolution = 64 × 64 matrix, slices = 28, thickness = 4 mm, gap = 1 mm, bandwidth = 2232 Hz/pixel]. The scan lasted for 478 s. 3D T1-weighted magnetization- prepared rapid gradient echo (MPRAGE) sagittal images were collected by using the following parameters: TR/TE/inversion time (TI)/FA = 1900 ms/2.2 ms/900 ms/9°, resolution = 256 × 256 matrix, slices = 176, thickness = 1 mm.

Unless otherwise stated, all preprocessing procedures were conducted using the toolbox of Data Processing Assistant for Resting-State fMRI (DPARSF) (Chao-Gan and Yu-Feng, 2010). Particularly, the first 10 volumes of the functional images were discarded for the signal equilibrium and participants' adaptation to the scanning noise. The remaining 229 fMRI images were first corrected for within-scan acquisition time differences between slices and then realigned to the first volume to correct for inter-scan head motions. No participant had a head motion of more than 1.5 mm maximum displacement in any of the *x, y*, or *z* directions and 1.5° of any angular motion throughout the course of the scan. The individual structural image was co-registered to the mean functional image after motion correction using a linear transformation. The transformed structural images were then segmented into gray matter (GM), white matter (WM), and cerebrospinal fluid (CSF) by using a unified segmentation algorithm (Ashburner and Friston, 2005). The motion corrected functional volumes were spatially normalized to the Montreal Neurological Institute (MNI) space and re-sampled to 3 mm isotropic voxels using the normalization parameters estimated during unified segmentation.

Subsequently, the functional images were spatially smoothed with a Gaussian kernel of 6 × 6 × 6 mm^3^ full width at half maximum (FWHM) to decrease spatial noise. Following this, temporal filtering (0.01–0.08 Hz) was applied to the time series of each voxel to reduce the effect of low-frequency drifts and high-frequency noise. To further reduce the effects of confounding factors, we also used a linear regression process to further remove the effects of head motion and other possible sources of artifacts (Fox et al., 2005): (1) six motion parameters; (2) whole-brain signal averaged over the entire brain; 3) linear drift.

## Methodology

### Overview

**Figure 1:**
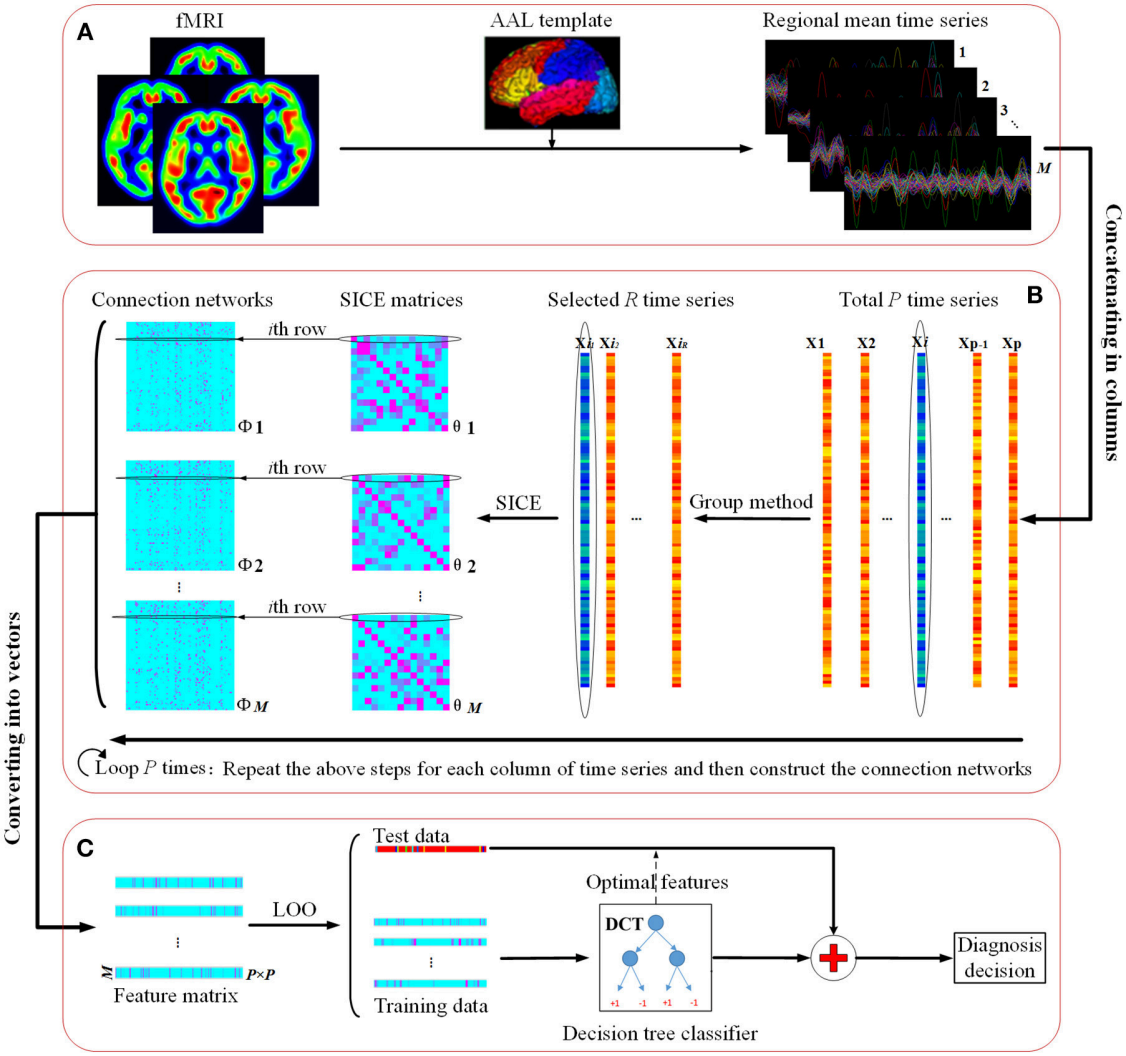
The flowchart of the proposed classification framework. **(A)** fMRI image preprocessing. **(B)** Sparse functional connectivity network construction. **(C)** Classification and decision.

Figure 1 illustrates the flowchart of our proposed classification framework, which includes the image preprocessing, sparse network construction and the evaluation of the classification performance. Specifically, fMRI images are first parceled into 90 regions-of-interest (ROIs) based on the Automated Anatomical Labeling (AAL) template (Tzourio-Mazoyer et al., 2002), and thus regional mean time series are acquired for each ROI. Second, the most discriminative brain regions subset for each ROI is detected by using the proposed Group-constrained topology structure detection algorithm, where ROIs subset with the smallest Bayesian information criterion (BIC) score is finally selected as the most discriminative brain regions. Furthermore, a functional connectivity network for each subject is constructed by using the SICE method. Finally, a LOOCV framework, together with an optimal DCT classifier, is implemented to evaluate the generalization performance of classification tasks.

### Construction of sparse functional network and feature extraction

The Group-constrained topology structure detection algorithm is first used to detect the most discriminative ROIs subset for each brain region. Then, a detailed description of the SICE method is presented to construct the functional connectivity network for each subject and a feature matrix is further built for the classification. Particularly, a non-sparse brain network construction method is also given as a comparison with the proposed method.

#### Group-constrained topology structure detection

Suppose that there are *M* subjects (*M* = 55), each subject includes the total of *P* ROIs (*P* = 90), and each ROI time series includes *n*_*t*_ time points (*n*_*t*_ = 229), the Group-constrained topology structure detection algorithm is first used to select the most discriminative ROIs by solving the following optimization rule (Wee et al., 2014):

(1) $${\hat{\beta }}_{p}=argmin\left(\sum_{m=1}^{M}{\left\|x_{p}^{m}-X_{p}^{m}\beta_{p}^{m}\right\|}_{2}^{2}+\varphi {\left\|\beta_{p}\right\|}_{2,1}\right),$$

where $x_{p}^{m}={\left[x_{p}^{m}(1),\;x_{p}^{m}(2),\;\ldots ,x_{p}^{m}(n_{t})\right]}^{T}(p=1,2,\ldots ,P)$ denotes the fMRI time series for the *p*th ROI of the *m*th subject, $X_{p}^{m}=[x_{1}^{m},x_{2}^{m},\ldots ,x_{p-1}^{m},\;x_{p+1}^{m},\ldots ,\;x_{P}^{m}]$ is a matrix including all ROIs time series of the *m*th subject except the *p*th ROI, $\beta_{p}^{m}={\left[\beta_{p,1}^{m},\;\beta_{p,2}^{m},\;\ldots ,\;\beta_{p,p-1}^{m},\beta_{p,p+1}^{m},\;\ldots ,\beta_{p,P}^{m}\right]}^{T}$ is the coefficient vector for the *p*th ROI of the *m*th subject, $\beta_{p}=[\beta_{p}^{1},\;\beta_{p}^{2},\ldots ,\beta_{p}^{M}]$ is the coefficient matrix for the *p*th ROI of all subject, $||•|{|}_{2}^{2}$ means the square of the *l*_2_-norm of a vector, i.e., the quadratic sum of all the elements in the vector, and ||•||_2, 1_ is the summation of the *l*_2_-norm of each row in the matrix. The nonzero elements in the coefficient matrix ${\hat{\beta }}_{p}$ are corresponding to the most discriminative ROIs for the *p*th ROI. The *l*_2, 1_-norm in Equation (1) encourages an identical optimal ROIs subset for the *p*th ROI among subjects. The regularization parameter φ∈(0, 1) controls the trade-off between fidelity term $\sum_{m=1}^{M}{\left\|x_{p}^{m}-X_{p}^{m}\beta_{p}^{m}\right\|}_{2}^{2}$ and regularization term ||***β***_*p*_||_2, 1_. Specifically, the Bayesian Information Criterion (BIC) (Schwarz, 1978) is used to optimize the regularization parameter φ:

(2) $$\begin{array}{r} BIC\left(\varphi \right)=-2L\left(\beta \left(\varphi \right)\right)+d\left(\varphi \right)\log n_{t} \end{array}$$

where *L*(β(φ)) is the log-likelihood function and *d*(φ) was the degree of freedom. The BIC score is defined by:

(3) $$\begin{array}{r} BIC=\overset{M}{\underset{m=1}{\sum }}\ln ESS\left(m\right)+\frac{d\ln\;n_{t}}{n_{t}} \end{array}$$

where *ESS*(*m*) is the residual sum of squares for the *m*th subject and *d* denotes the number of nonzero coefficients in the matrix ${\hat{\beta }}_{p}$. For each φ, a BIC score is calculated by using Equation (3) and the subset of ROIs with smallest BIC score are finally determined as the most discriminative brain regions for the *p*th ROI. Thus, an optimal ROI subset ***E***_*p*_ for the *p*th ROI is obtained. Repeat the same steps for all ROIs, the most discriminative ROIs subset for each ROI can be obtained.

#### Sparse network construction

Many methods have been proposed to construct the human brain connectivity networks, among which the sparse representation-based method (Tibshirani et al., 2005; Wright et al., 2009; Wee et al., 2016) and the correlation-based method (Wee et al., 2012; Jie et al., 2014) are two popular approaches. Compared with the correlation-based method, the sparse representation-based method usually has much better discriminability due to the small-world properties and scale-free attributes of human brain connectivity networks. Therefore, a sparse representation-based method (SICE) in this study was adopted to construct the human brain networks. In addition, a non-sparse network was also used for a comparison to show the advantages of our proposed method.

##### Non-sparse network construction

Partial correlation matrix Π was used as the comparison in this study, which measures the relationship between two time series after excluding the effects of all other time series. Specifically, if the correlation matrix Σ is positively defined and therefore invertible, then partial correlation matrix Π can be acquired from the full inverse covariance matrix Σ^−1^ by using the following formula:

(4) $$\begin{array}{r} \Pi_{ij}=\frac{-{\left(\Sigma^{-1}\right)}_{ij}}{\sqrt{{\left(\Sigma^{-1}\right)}_{ii}{\left(\Sigma^{-1}\right)}_{jj}}}\;\left(i\neq j\right), \end{array}$$

Considering the fact that the length of the regional mean time series (229 points in this study) is larger than the number of brain regions (i.e., 90 ROIs), it is capable of calculating the full inverse covariance matrix Σ^−1^ from the time series data directly. Firstly, we computed the covariance matrix Σ for each pair of brain regions using the following formula:

(5) $$\Sigma_{pq}^{m}=\frac{\sum_{i=1}^{n_{t}}\left(x_{p}^{m}\left(i\right)-x_{p}^{m})(x_{q}^{\bar{m}}\left(i\right)-x_{q}^{\bar{m}}\right)}{n_{t}-1},$$

where $\Sigma_{pq}^{m}$ is the covariance between the time series for the *p*th ROI ${\text{x}}_{p}^{m}$ and the *q*th ROI ${\text{x}}_{q}^{m}$ from the *m*th subject, $\bar{x_{p}^{m}}$ and $\bar{x_{q}^{m}}$ denote the mean of the ${\text{x}}_{p}^{m}$ and ${\text{x}}_{q}^{m}$, respectively. Then, the full inverse covariance matrix Σ^−1^ is directly computed from the covariance matrix Σ.

##### Sparse network construction

The SICE method, which finds a sparse inverse covariance matrix by imposing a “sparsity” constraint on the maximum likelihood estimation of the inverse matrix, is used to construct the sparse functional brain sub-network.

Suppose the optimal ROIs time-series subset for the *p*th ROI selected in section Group-Constrained Topology Structure Detection is $E_{p}=\left[y_{1}^{p},\;y_{2}^{p},\ldots ,y_{R}^{p}\right]$ (*n*_*t*_ time-points × *R* regions), where *y*_*r*_ denotes the fMRI time series for the *r*th selected ROI. The expanded-***E***_*p*_ containing the target brain region (*p*th ROI) can be expressed as ${\tilde{E}}_{p}=\left[y_{0}^{p},E_{p}\right]=[y_{0}^{p},y_{1}^{p},\;y_{2}^{p},\ldots ,y_{R}^{p}]$, where ${\text{y}}_{0}^{p}$ is the time series for the *p*th ROI.

The SICE method tries to find an estimate for the inverse covariance matrix $\hat{\Theta }$ for ${\tilde{E}}_{p}$ by solving the following optimization:

(6) $$\hat{\Theta }=argmin_{\Theta >0}(-\log\left(\det\left(\Theta \right)\right)+tr\left(S\Theta \right)+\lambda {\left\|\Theta \right\|}_{1}),$$

where det(•) and *tr*(•) denote the determinant and trace of a matrix, **S** is the sample-based covariance matrix for ${\tilde{E}}_{p}$, ||•||_1_ is the sum of absolute values of all the entries in a matrix, and λ is the regularization parameter, respectively. Considering a trade-off between the likelihood estimation and the regularization term ||***Θ***||_1_, the Equation (6) can also be written as follows:

(7) $$\begin{array}{l} \hat{\Theta }=argmin_{\Theta >0}(-\log\left(\det\left(\Theta \right)\right)+tr\left(S\Theta \right)),\\ \;subject\;to\;{\left\|\Theta \right\|}_{1}\leq \varepsilon , \end{array}$$

where ε(ε>0) is reversely related to λ. When ε is large enough (i.e., small λ), the constraint ||***Θ***||_1_ ≤ ε has little effect and the SICE is just a usual Maximum likelihood estimation. Conversely, when ε is small enough (i.e., large λ), the SICE can produce a shrunken estimate for ***Θ***, and effectively set certain coefficients in $\hat{\Theta }$ exactly to zero. For example, if the *ij*th element in the connection matrix $\hat{\Theta }$ is zero, the region *i* and *j* are conditionally non-connection. Conversely, the nonzero elements in $\hat{\Theta }$ indicate the connection between two regions of ROI. Specifically, due to the time series for the target brain region is the first column of ${\tilde{E}}_{p}$. Thus, the first column/row of $\hat{\Theta }$ denotes the connection between the target brain region and its most discriminative ROIs subset. Therefore, the functional connectivity network can be constructed by using the sparse inverse covariance matrix $\hat{\Theta }$.

#### Feature extraction

For the *p*th ROI, the Group-constrained topology structure detection algorithm is performed on all the other ROIs' time series to select the ROIs set ***E***_*p*_ that included the most discriminative brain regions. Then, these ROIs time series together with the target ROI time series are used to construct the functional connectivity networks based on the SICE method. Specifically, the constructed functional connectivity networks $\Theta_{m}^{p}$, where *m* = 1, 2, …, *M*, with the total number of subjects *M*, can be regarded as sub-networks of the whole brain from the *p*th ROI, since all brain regions used in ROIs set ***E***_*p*_ are connected with the *p*th ROI. Additionally, we can take the first column of $\Theta_{m}^{p}$ as the weight vector, $v_{m}^{p}$, that quantifies the degree of the influence of other ROIs to the *p*th ROI. Considering that the dimensions of the connection matrix ***Θ***_*m*_ is *P*×*P*, with the total number of ROIs *P*, we put the elements of $v_{m}^{p}$ in the corresponding positions of the *p*th column of ***Θ***_*m*_ according to the ROI indexes.

Repeating the above processes *M* times, we can obtain a connection matrix ***Θ*** for each subject, with the *p*th column denoting the connections between the *p*th ROI and the other ROIs. Then, the connection matrix ***Θ*** is converted into a feature vector with 8,100 features (90 × 90 ROIs), where the element represented the connectivity strength between two ROIs for the given subject. Finally, a *N*_*subjects*_×*N*_*edges*_ feature matrix was obtained, where *N*_*subjects*_ is the number of subjects, and *N*_*edges*_ denotes the number of the features. A detailed procedure of the feature extraction is given in part B of Figure 1.

### Classification and validation

**Figure 2:**
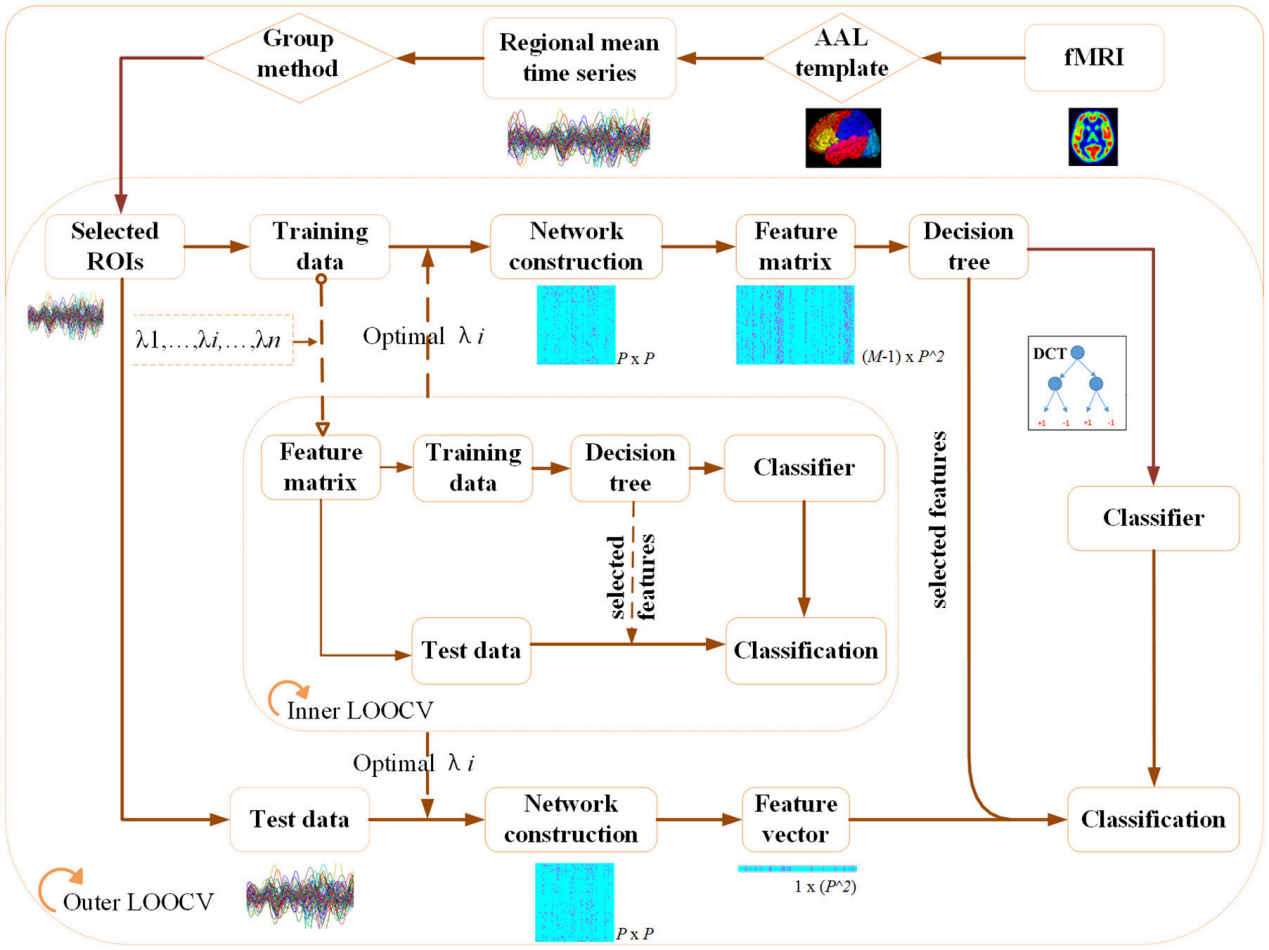
The nested LOOCV classification framework.

Considering the limited sample size, the following nested LOOCV scheme (Figure 2) is adopted to evaluate the classification performance of our proposed method. The LOOCV is reported as an effective method to obtain a reliable accuracy estimate for the small sample size classification (Wong, 2015). In the outer LOOCV loop, suppose the whole dataset consists of *M* subjects (*M* = 55 in this study), one subject is first left out for testing and the remaining *M*−1 subjects are used for training the classifier. The above procedures will be repeated *M* times and each time a different subject will be left out for testing the performance of the classifier, which is trained based on the remaining *M*−1 subjects. In this way, each subject is used as the test subject for one time and *M* classification results will be obtained. Finally, the average cross-validation classification accuracy is achieved.

In each repeat of the outer loop, we further performed an inner LOOCV loop on the *M*−1 training data to optimize the hyper-parameter λ which is used for the network construction. Specifically, the *M*−1 training subjects will be further separated into *M*−2 training subjects and one testing subject. Repeating the inner loop procedure *M*−1 times and each time a different subject will be left out for testing. Finally, the parameter λ with the maximum classification accuracy in the inner loop is transmitted to the outer loop for classifying the AD from NC.

## Results

### Comparison of classification performance

**Figure 3:**
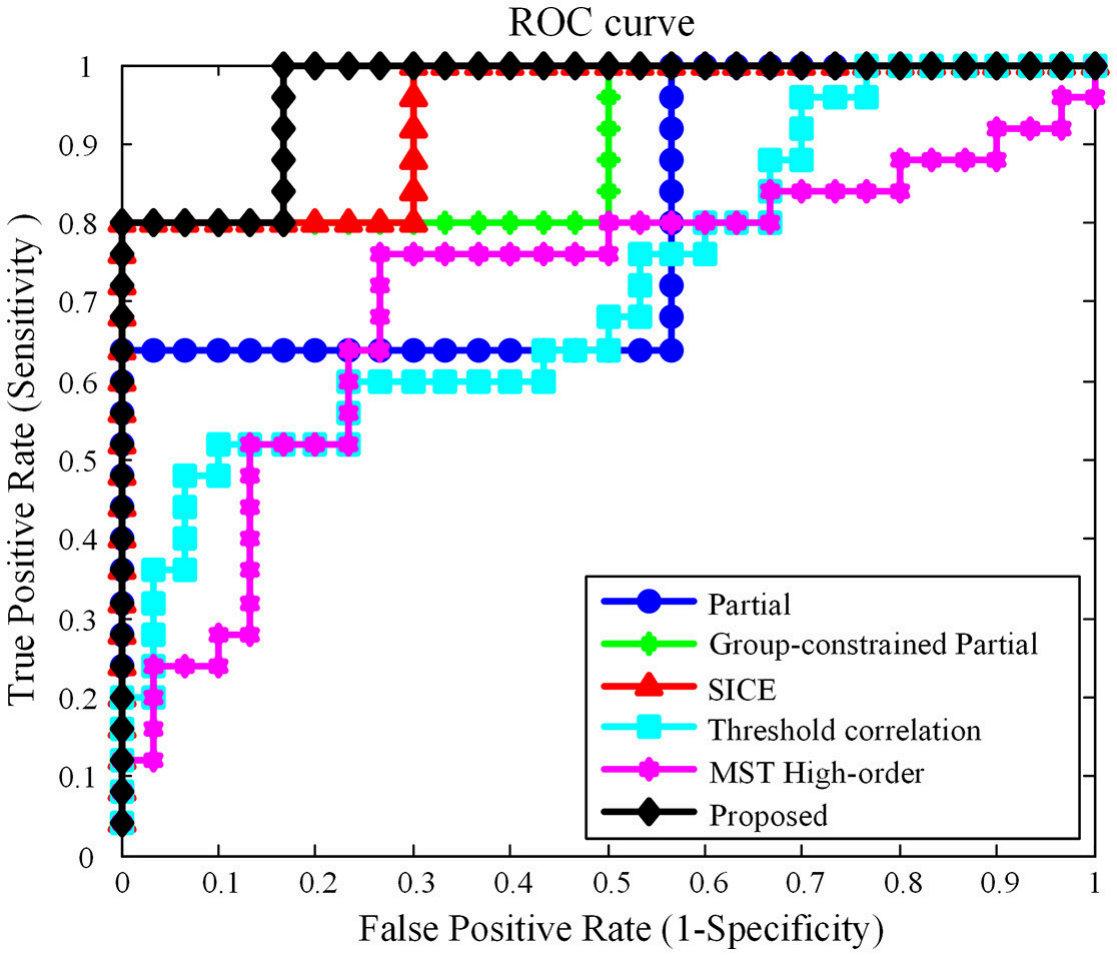
ROC curves of different classification methods.

In this work, using the same dataset, the proposed Group-constrained topology structure detection + SICE (Group-constrained SICE) method is compared with other related works including the Partial method, the Group-constrained topology structure detection + Partial (Group-constrained Partial) method, and the SICE method. We further compare the performance of our proposed framework with the recent existing methods for AD classification, including the threshold correlation method (Khazaee et al., 2016) and the minimum spanning tree (MST) high-order method (Guo et al., 2017). In the threshold correlation approach (Khazaee et al., 2016), the brain network is constructed using the Pearson correlation coefficient to calculate the functional connectivity of all pairs of brain regions. Then, the weak connections are removed by maximizing the global cost efficiency (GCE) of the network. In the MST high-order approach (Guo et al., 2017), a high-order network is first constructed based on the sliding window and the Pearson correlation coefficient. Then, the high-order network is pruned by the MST method to construct the MST high-order network. The classification accuracy (ACC), sensitivity (SEN) (Wang et al., 2017), specificity (SPE), and AUC (Li et al., 2018) are used to measure the classification performance of different classification methods. The classification results are summarized in Table 1. Figure 3 presents the ROC (Li et al., 2017) curves of different classification methods.

**Table 1:** The classification performance of different classification methods.

**Method**	**ACC (%)**	**SEN (%)**	**SPE (%)**	**AUC**
Partial	58.18	64.00	53.33	0.7960
Group-constrained Partial	63.64	80.00	50.00	0.9000
SICE	74.55	80.00	70.00	0.9400
Threshold correlation	69.09	60.00	76.67	0.7200
MST High-order	74.55	76.00	73.33	0.7067
**Group-constrained SICE**	**81.82**	**80.00**	**83.33**	**0.9667**

*Where bold values indicate the best results*.

As shown in Table 1, our proposed classification method yielded a classification accuracy of 81.82% with a sensitivity of 80.0% and a specificity of 83.33%, while the best accuracy was only reached to 74.55% by the direct SICE method. The cross-validation estimation of the generalization performance showed 0.9667 of AUC with the proposed method, indicating the classification power of our proposed scheme.

### The optimal lambda

**Figure 4:**
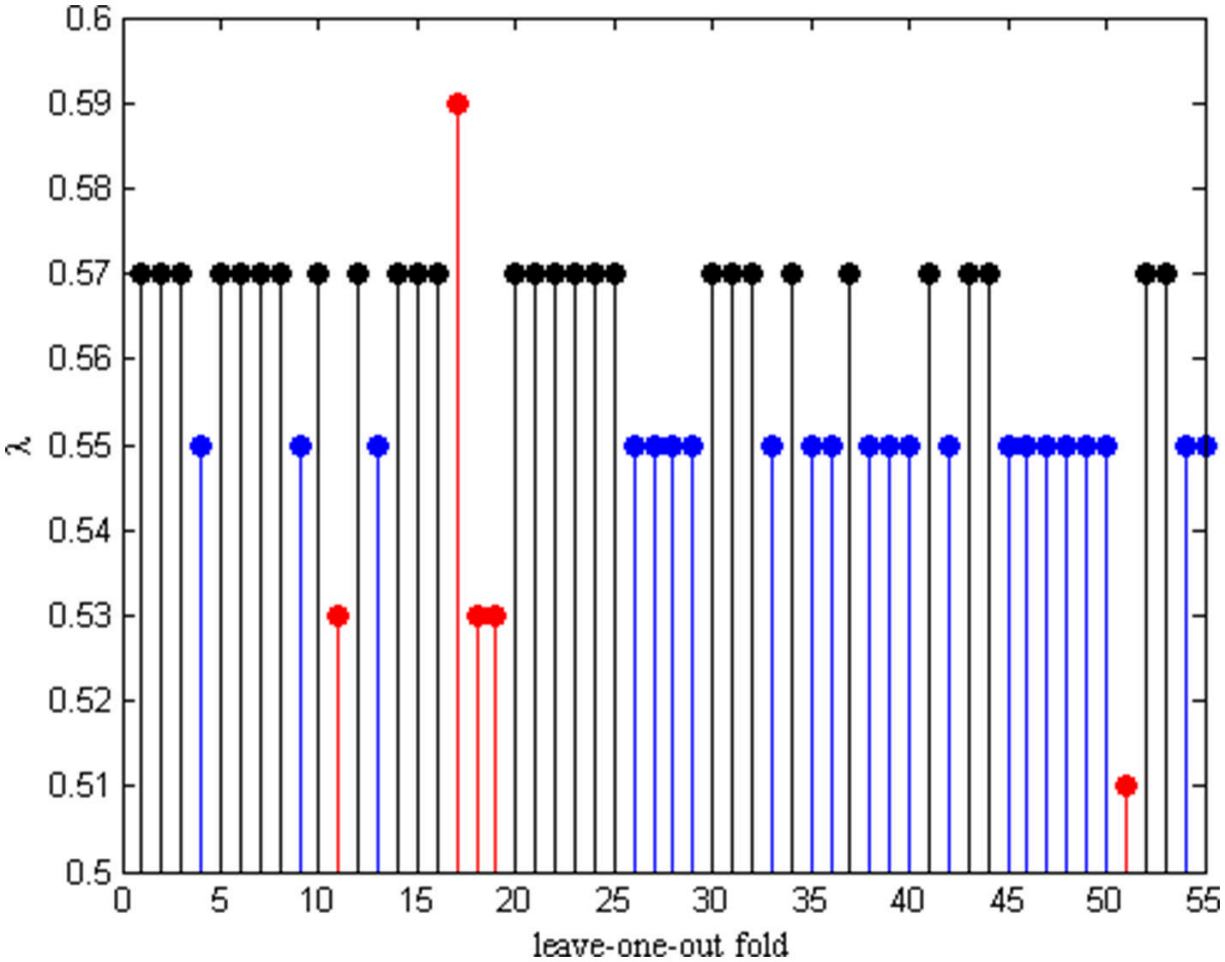
The optimal λ in each LOO-fold.

In order to hunt for the optimal parameter λ, an inner LOOCV framework is exploited to determine λ in each leave-one-out fold. Therefore, different λ will be selected from the λ pool each time. The optimal λ in each fold is given in Figure 4. We can see that λ = 0.55 and λ = 0.57 are selected the 22 frequency times and 28 frequency times from the total number of 55 subjects, respectively. These results indicates that the functional connectivity networks based on λ = 0.55 and λ = 0.57 constructed have better discriminability between AD and NC.

### Computational complexity and convergence

The computational complexity of the proposed method is evaluated by the computation time. All timings are carried out on an Intel Core i7-4790 3.60GHz processor. Each subsection of the methods has been run 10 times and the mean of the computation time is shown in Table 2.

**Table 2:** The computation times (seconds) of all compared frameworks.

**Method**	**Group**	**Partial**	**SICE**	**Other**	**Total**
Partial	–	2.307	–	–	2.307
Group-constrained Partial	1,115.674	2.274	–	–	1,117.948
SICE	–	–	244.738	–	244.738
Threshold correlation	–	–	–	1,156.468	1,156.468
MST High-order	–	–	–	7,415.385	7,415.385
Group-constrained SICE	1,115.674	–	16.907	–	1,132.581

*Where each column represents the computation time of the subsection of the method*.

It is obvious that the computation time of Partial method is significantly shorter than other methods. However, with a classification accuracy of 58.18%, the Partial correlation is not considered to be an effective method for AD classification. The computation times of the Group-constrained Partial method and Group-constrained SICE method (the proposed method) are similar, where the main computation time comes from the Group-constrained topology structure detection algorithm. Although the computation load of the proposed method is heavier than the SICE method, there is no significant of magnitude difference between them, and the computational issue becomes less critical when the performance of PC develops rapidly. Furthermore, applying the SICE at an individual level will inevitably cause the inter-subject variability, thus reducing the generalization performance. Therefore, it is crucial to adopt the Group-constrained method, which encourages an identical network topology across subjects, to overcome the limitation of SCIE. Interestingly, the computation time of the SICE in the proposed method is shorter than that of using SICE alone. This phenomenon can be interpreted as that the Group-constrained topology structure detection algorithm is adopted to select the most relevant brain regions, reducing the number of brain regions calculated in the SICE. The computation time of the threshold correlation method (Khazaee et al., 2016) is slightly longer than that of our proposed method. Due to the large scale of the high-order network, the computation complexity of the MST high-order method (Guo et al., 2017) is significantly higher than other methods.

**Figure 5:**
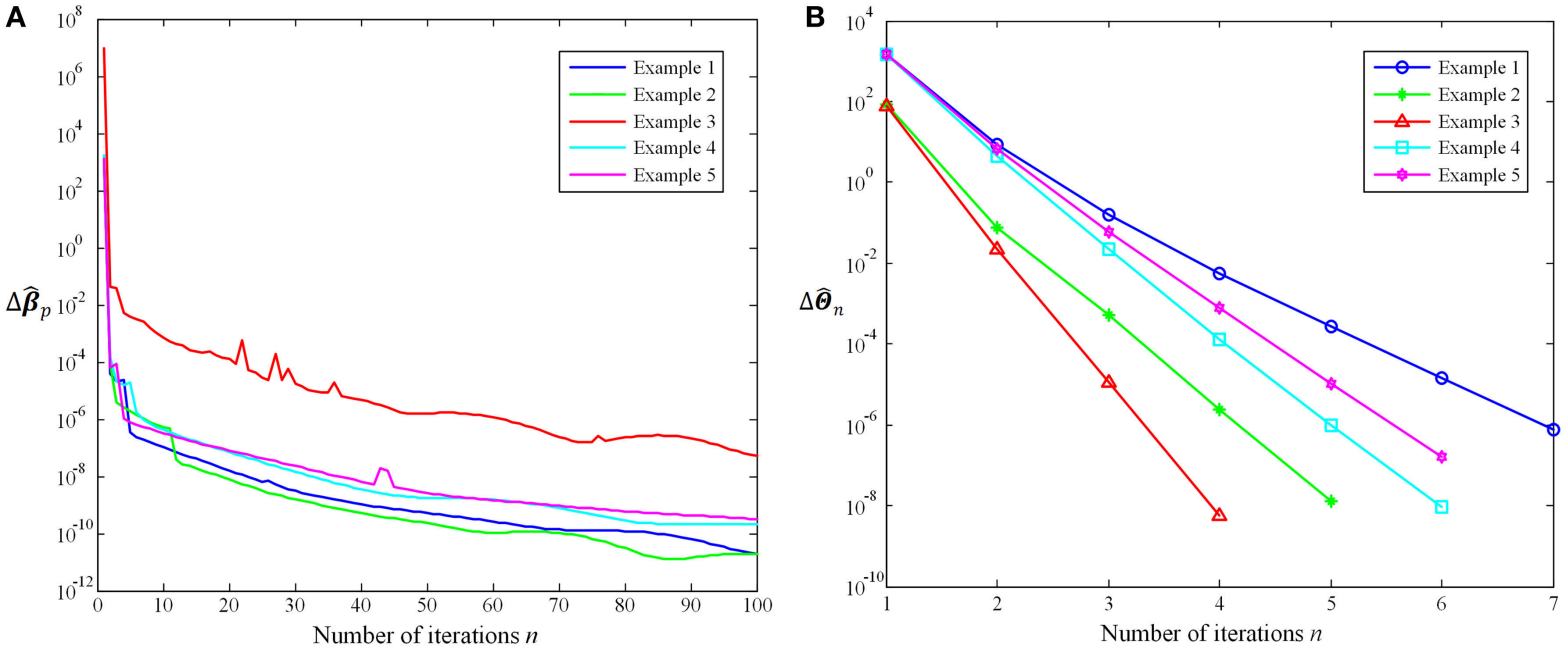
**(A)** $\Delta {\hat{\beta }}_{p}$ trend with the number of iterations *n*. **(B)** $\Delta {\hat{\Theta }}_{n}$ trend with the number of iterations *n*.

In this work, we further evaluate the proposed method convergence based on the trend of coefficients variation with the number of iterations. On the one hand, the iteration of the Group-constrained topology structure detection algorithm will be repeated until *n* = 100, where *n* is the number of iterations. $\Delta {\hat{\beta }}_{p}=Frobenius\;norm\;of\;{\left({\hat{\beta }}_{p}\right)}_{n}-{\left({\hat{\beta }}_{p}\right)}_{n-1}$ describes how much the coefficient matrix changes in the *n*th iteration. In the 100th iterations, $\max\left(\Delta {\hat{\beta }}_{p}\right)=1.17\times 10^{-6}$ and $mean\left(\Delta {\hat{\beta }}_{p}\right)=5.25\times 10^{-9}$. Figure 5A shows the $\Delta {\hat{\beta }}_{p}$ trend with the number of iterations *n* using five examples. On the other hand, the iteration of the SICE will stop when $\Delta {\hat{\Theta }}_{n}$ < 10^−6^, where $\Delta {\hat{\Theta }}_{n}$ is the sum of absolute values of ${\hat{\Theta }}_{n}-{\hat{\Theta }}_{n-1}$. The average number of iterations is 5.57 and the maximum number is 13. Figure 5B shows the $\Delta {\hat{\Theta }}_{n}$ trend with the number of iterations *n* using five examples. It is obvious that as the number of iterations *n* increases, the variation of coefficients ($\Delta {\hat{\beta }}_{p}$ and $\Delta {\hat{\Theta }}_{n}$) decreases rapidly. The experimental results suggest that the proposed method is convergent and has a high rate of convergence.

### Brain regions involved in classification

**Figure 6:**
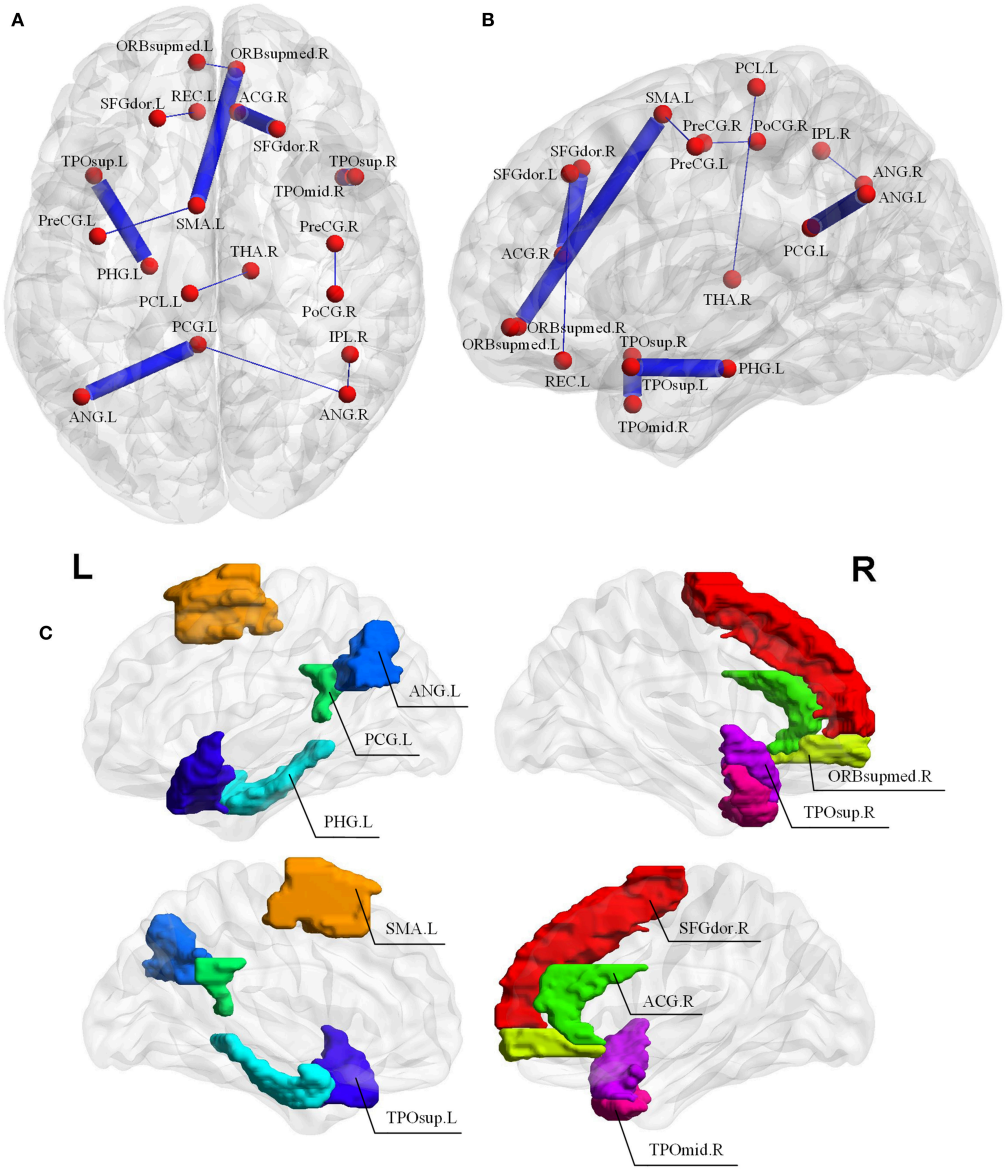
**(A,B)** Selected connections in the LOOCV folds. The width of edges connecting two ROIs corresponds to the degree of discrimination. **(C)** The discriminative brain regions selected by our proposed method for AD classification. The corresponding ROI names of the abbreviations are as follows: TPOsup.R, Temporal_Pole_Sup_R; TPOmid.R, Temporal_Pole_Mid_R; PHG.L, ParaHippocampal_L; TPOsup.L, Temporal_Pole_Sup_L; CG.L, Cingulum_Post_L; ANG.L, Angular_L; SMA.L, Supp_Motor_Area_L; ORBsupmed.R, Frontal_Med_Orb_R; SFGdor.R, Frontal_Sup_R; ACG.R, Cingulum_Ant_R; PreCG.L, Precentral_L; PreCG.R, Precentral_R; PoCG.R, Postcentral_R; REC.L, Rectus_L; ORBsupmed.L, Frontal_Mid_Orb_L; IPL.R, Parietal_Inf_R; ANG.R, Angular_R; THA.R, Thalamus_R; SFGdor.L, Frontal_Sup_L; PCL.L, Paracentral_Lobule_L.

Since the nested LOOCV is used to evaluate the performance of the proposed framework, different optimal feature (connection between two ROIs) subsets are selected for AD classification. Thus, those connections with the highest selected frequency times in the nested LOOCV are regarded as the most discriminative features for AD classification. Figures 6A,B show the selected connections among all LOO folds. A detailed feature index and its frequency times selected are summarized in Table 3. For example, the interconnection between Temporal_Pole_Sup_R and Temporal_ Pole_Mid_R is selected 55 frequency times among all LOO folds, and showed very strong discriminative strength in AD diagnosis. In addition, the following four interconnections including ParaHippocampal L-Temporal_ Pole_Sup_L, Cingulum_Post_L-Angular_L, Supp_Motor_ Area_L-Frontal_Med_Orb_R, and Frontal_ Sup_R-Cingulum_Ant_R are all selected with over 50 frequency times. These connections have much higher selected frequency times than others, thus can be regarded as effective biomarkers for identifying AD from healthy elderly.

**Table 3:** Selected connections by the proposed classification framework.

**No**.	**Functional connectivity**	**No. of frequency**
1	Temporal_Pole_Sup_R----Temporal_Pole_Mid_R	55
2	ParaHippocampal_L---- Temporal_Pole_Sup_L	53
3	Cingulum_Post_L----Angular_L	51
4	Supp_Motor_Area_L----Frontal_Med_Orb_R	51
5	Frontal_Sup_R----Cingulum_Ant_R	51
6	Precentral_L----Supp_Motor_Area_L	2
7	Precentral_R----Postcentral_R	1
8	Frontal_Sup_L----Rectus_L	1
9	Frontal_Med_Orb_L----Frontal_Med_Orb_R	1
10	Parietal_Inf_R----Angular_R	1
11	Angular_R----Cingulum_Post_L	1
12	Thalamus_R ----Paracentral_Lobule_L	1

It should be noted that the brain regions involved in the significant abnormal connectivity pathways (with over 50 selected frequency times) are located mainly within the default mode network (DMN), including Temporal_Pole_Sup_R, Temporal_Pole_Sup_L, Temporal_Pole_ Mid_R, ParaHippocampal_L, Cingulum_Post_L, Angular_L, Supp_Motor_Area_L, Frontal_ Med_Orb_R, Frontal_Sup_R, Cingulum_Ant_R. These brain regions, listed in Table 4 and displayed in Figure 6C, are reported as highly associated with AD pathology (Rose et al., 2006; Matsuda, 2013; Salvatore et al., 2015; Scheff et al., 2015; Xu et al., 2016; Loewenstein et al., 2018).

**Table 4:** The discriminative brain regions.

**No**.	**ROI index**	**ROI abbr**.	**ROI name**	**References**
1	84	TPOsup.R	Temporal_Pole_Sup_R	Salvatore et al., 2015
2	83	TPOsup.L	Temporal_Pole_Sup_L	Salvatore et al., 2015
3	88	TPOmid.R	Temporal_Pole_Mid_R	Salvatore et al., 2015
4	39	PHG.L	ParaHippocampal_L	Matsuda, 2013
5	35	PCG.L	Cingulum_Post_L	Scheff et al., 2015
6	65	ANG.L	Angular_L	Xu et al., 2016
7	19	SMA.L	Supp_Motor_Area_L	Rose et al., 2006
8	26	ORBsupmed.R	Frontal_Med_Orb_R	Loewenstein et al., 2018
9	4	SFGdor.R	Frontal_Sup_R	Salvatore et al., 2015
10	32	ACG.R	Cingulum_Ant_R	Salvatore et al., 2015

## Discussion

### Classification performance

This paper proposed a new classification framework based on fMRI time series for diagnosing AD patients. The combination of the Group-constrained topology structure detection algorithm with the SICE, where a nested LOOCV method is employed to optimize the regularization parameter, are designed to construct the efficient functional brain sub-networks. Then, an optimal DCT classifier is trained for classifying AD from NC based on the optimal brain sub-networks. Experimental results demonstrate the effectiveness of the proposed method.

Specifically, in contrast to the other methods, experimental results show that the proposed classification method has at least 7.27% improvement of the diagnosis accuracy. The classification result indicates that the sparse-based method is more appropriate for brain network construction than the traditional fully-connected correlation-based networks, which may contain a large number of spurious or insignificant connections among ROIs. It was also found that both the SICE and Partial method aided by the Group-constrained topology structure detection method can improve the classification performance. This may be due to the group-constrained topology structure detection algorithm encourages an identical network topology across subjects, minimizing the inter-subject variability problem which degrades generalization performance of trained classifiers.

### The most discriminative brain regions and connections

The top 5 brain connections listed in Table 3 have much higher selected frequency times than others, which may serve as the more promising connectivity-based biomarker for AD diagnosis. These results are totally consistent with previous findings, and added new findings to the disconnection hypothesis of the AD (Delbeuck et al., 2003; Lacalle-Aurioles et al., 2016). The brain regions identified in the top 5 connections are frequently reported as highly associated with the AD pathology. For example, Scheff et al. (2015) reported that the AD patients showed a significant decline in synaptic numbers in the Cingulum_Post (posterior cingulate gyrus) compared to healthy elderly. Furthermore, these brain regions mainly belong to DMN (Raichle et al., 2001), which is one of the earliest pathological sites of the AD (Greicius et al., 2004). The current findings are in line with the results reported in the related literature that both the impairment and compensation coexist in DMN of AD (Sorg et al., 2007; Liang et al., 2014). Given the disconnection within DMN can also be detected in the mild cognitive impairment (MCI) stage of the AD (Qi et al., 2010; Wang et al., 2012), it is expected that the proposed method may also be applied to the diagnosis of MCI patients. This will be discussed in our future studies.

### Methodological limitations

There are still two limitations in this study. One limitation is that we set the tuning parameter λ of the SICE for identifying for different subjects, which may affect the classification performance, since the optimal parameter may vary across subjects due to individual differences. To overcome this issue, one possible solution is to optimize the parameter λ for each subject using the BIC method. In this way, we can construct optimal connectivity networks for each subject and this will be investigated in our future works. Another limitation lies in the brain atlas used in the MRI data analysis. Given the participants in this study are from Chinese populations as well as the significant morphological difference between Chinese and Caucasian population (Tang et al., 2010), the statistical Chinese brain atlas (Liang et al., 2015) rather than the Caucasian brain atlas (as implemented in SPM8) should be used during the image segmentation and registration. This may be helpful for extracting the more exact MRI features of the two groups of participants, which thus may improve the diagnostic accuracy of AD patients.

## Conclusion

In this paper, we have proposed a novel sub-network based classification framework to construct brain functional sub-connectivity and explore its diagnostic power in distinguishing AD patients from NC. Different from the method based on the whole-brain level, we constructed brain sub-networks with the most discriminative brain regions. Experimental results have verified the validity of the proposed classification framework.

## Ethical approval

All procedures performed in studies involving human participants were in accordance with the ethical standards of the institutional and/or national research committee and with the 1964 Helsinki declaration and its later amendments or comparable ethical standards.

## Informed consent

Informed consent was obtained from all individual participants included in the study.

## Author contributions

YL designed the framework, explained the results and wrote the paper. JL supplemented the experiment and revised the paper. JH made the program and wrote the paper. ZL made the program and wrote the paper. PL designed the study, explained the results, and wrote the paper.

### Conflict of interest statement

The authors declare that the research was conducted in the absence of any commercial or financial relationships that could be construed as a potential conflict of interest.
